# If you feel you can’t, you won’t: the role of subjective and objective cognitive competence on psychosocial functioning in depression

**DOI:** 10.1192/j.eurpsy.2023.2454

**Published:** 2023-10-19

**Authors:** Muriel Vicent-Gil, Joan Trujols, Maria Serra-Blasco, Guillem Navarra-Ventura, Dolors Puigdemont, Carlo Alemany, Sara Crivillés, Javier de Diego-Adeliño, Narcís Cardoner, Maria J. Portella

**Affiliations:** 1 Sant Pau Mental Health Research Group, Institut d’Investigació Biomèdica Sant Pau (IIB-Sant Pau), Hospital de la Santa Creu i Sant Pau, Centro de Investigación Biomédica en Red de Salud Mental, Instituto de Salud Carlos III (CIBERSAM-ISCIII), Universitat Autònoma de Barcelona (UAB), Barcelona, Spain; 2 eHealth ICOnnecta’t Program and Psycho-Oncology Service, Institut Català d’Oncologia, L’Hospitalet de Llobregat, Spain; 3 Institut d’Investigació Sanitària Illes Balears (IdISBa), Hospital Universitari Son Espases, Palma, Spain; 4 Institut Universitari d’Investigació en Ciències de la Salut (IUNICS), Universitat de les Illes Balears, Palma, Spain; 5 Centro de Investigación Biomédica en Red de Enfermedades Respiratorias, Instituto de Salud Carlos III (CIBERES-ISCIII), Madrid, Spain; 6 Department of Psychiatry and Legal Medicine, Institute of Neuroscience, Universitat Autònoma de Barcelona (UAB)

**Keywords:** cognition, depression, executive function, psychosocial functioning, self-appraisal

## Abstract

**Background:**

The purpose of this exploratory study is to examine the role of sociodemographic, clinical, and cognitive – both objective and subjective – factors in overall and in specific domains of psychosocial functioning, in patients with depression at different clinical states of the disease (remitted and non-remitted).

**Methods:**

A sample of 325 patients with major depressive disorder, 117 in remission and 208 in non-remission, were assessed with a semi-structured interview collecting sociodemographic, clinical, cognitive (with neuropsychological tests and the Perceived Deficit Questionnaire), and functional (Functioning Assessment Short Test) characteristics. Backward regression models were conducted to determine associations of global and specific areas of functioning with independent factors, for both clinical states.

**Results:**

Residual depressive symptomatology and self-appraisal of executive competence were significantly associated with psychosocial functioning in remitted patients, in overall and some subdomains of functioning, particularly cognitive and interpersonal areas. While depressive symptoms, executive deficits and self-appraisal of executive function were significantly related to functional outcomes in non-remitted patients, both in overall functioning and in most of subdomains.

**Discussion:**

This study evidences the strong association of one’s appraisal of executive competence with psychosocial functioning, together with depressive symptoms, both in remitted and non-remitted patients with depression. Therefore, to achieve full recovery, clinical management of patients should tackle not only the relief of core depressive symptoms, but also the cognitive ones, both those that are objectified with neuropsychological tests and those that are reported by the patients themselves.

## Introduction

Depression has always been associated with difficulties in daily functioning [1, 2]. According to the classification manual for mental disorders DSM-5, one of the diagnostic criteria is that depressive symptomatology causes clinically significant distress and/or impairment in social, work-related, or other important areas of one’s life. In this regard, there is scientific evidence upon the presence of psychosocial dysfunction in the acute phase of the illness [3, 4]. Different studies have shown how work performance, and family and social relationships are altered in depressed patients [5]. Contrary to expectations however, these functional difficulties also remain in stages of clinical improvement. Some studies have shown that patients with residual depressive symptoms may have the same functional impairment as patients in the severe phase of the disease. Therefore, psychosocial functioning remains impaired in periods of clinical remission [3, 6, 7].

To this point, the scientific research carried out in this field has focused on understanding which factors might be related to the functional impairment of patients suffering from depression. Clinical characteristics such as the stage of the illness (i.e., being in non-remission or in remission), showing residual depressive symptomatology, having a higher number of depressive episodes throughout the course of the illness, as well as an earlier age of illness onset, have been associated with a poorer psychosocial functioning [8–11]. Similarly, cognitive dysfunction seems to play a key role in functional impairment. On one side, some studies link objective difficulties in the cognitive domains of attention, verbal memory, and executive function with poorer functional outcomes [6, 12, 13]. On the other side, very few studies have taken into account patients’ appraisal of their own cognitive performance, and this little evidence only relates subjective measures of inattention to poorer work performance [9, 14]. However, it has not been studied whether these factors related to psychosocial functioning vary according to clinical stages, and this is indeed a matter of interest for clinical research as it allows the development of much more targeted interventions with the aim of achieving patients’ full recovery [4, 15].

Another issue to take into consideration is that in clinical practice not all depressed patients show the same pattern of functional impairment, meaning that patients could present a global impairment in daily activities, or impairment in one or some specific areas of psychosocial functioning. This functional heterogeneity has been little addressed in previous research [3, 5, 6], as most studies use total scores on questionnaires and scales as proxies of functional outcome [10]. In order to take current treatments a step further, it may be necessary to know which functional areas are particularly affected in each patient. Realizing whether there are difficulties at work, whether social activities are diminished, or whether social life cannot be enjoyed is crucial in the development of interventions tailored at each of the clinical stages of the disorder. Not only it is relevant to know what is affected, but also which factors may determine these functional differences in patients. For instance, cognitive symptoms have been widely associated with poorer psychosocial functioning in general [16, 17], but little evidence has been gathered on specific cognitive problems within specific psychosocial subdomains [18]. In this regard, both subjective cognitive complaints and objective neuropsychological performance may account for possible differences in specific functional difficulties.

To address the issue of functional heterogeneity in depression, the aim of the present exploratory cross-sectional study is to examine whether sociodemographic, clinical, and cognitive (objective and subjective) characteristics play a role in overall functioning and in the different areas of psychosocial functioning, in different clinical states of the disease (remitted and non-remitted patients).

## Materials and methods

### Participants

A total of 325 patients with a diagnosis of major depressive disorder (MDD; DSM-5 criteria) aged between 18 and 65 were recruited from the psychiatry departments of the Hospital de la Santa Creu i Sant Pau (Barcelona, Catalonia, Northeast Spain) and of the Hospital Universitari Parc Taulí (Sabadell, Catalonia, Northeast Spain). Patients were excluded if they met any of the following exclusion criteria: (i) an estimated intelligence quotient (IQ) lower than 85, (ii) any neurological disease or medical condition presenting cognitive deficits, and (iii) a history of substance abuse disorder except nicotine. The study followed the ethical principles of the Declaration of Helsinki and the principles of Good Clinical Practice, complied with current data protection law, and approved by the ethics committee of each hospital. All participants gave written informed consent after a comprehensive explanation of the study.

### Sociodemographic and clinical assessment

A semi-structured interview designed ad hoc was used to collect sociodemographic and clinical information from all participants, including age, sex, years of schooling, estimated IQ, age at illness onset, number of depressive episodes, and depressive symptomatology. Clinical variables were double-checked with clinical records and corrected if discrepant. Estimated IQ was assessed with the Vocabulary subtest of the Wechsler Adult Intelligence Scale version-IV (WAIS-IV; Wechsler, 2008). Hamilton Depression Rating Scale (HDRS-17) [19, 20] was used to evaluate depressive symptoms. Patients with a HDRS-17 score of 7 or less were considered to be in full clinical remission (remitted patients) and patients with scores of 8 or higher were classified as non-remitted (non-remitted patients).

### Cognitive assessment

#### Subjective cognitive function

The patients’ appraisal of their own cognitive functioning, referred as subjective cognition, was assessed by means of the Perceived Deficit Questionnaire (PDQ-20) [21–23]. The PDQ-20 is a 20-item self-report measure of cognitive dysfunction that assesses the most affected cognitive domains: attention, retrospective memory, prospective memory, and planning and organization. Each item is rated from 0 (never) to 5 (almost always); so higher scores indicate a higher perception of suffering from cognitive deficits.

#### Objective cognitive function

Objective cognition was measured with a battery of neuropsychological tests covering the domains of attention, verbal memory, and executive function, to assemble the same cognitive domains included in the PDQ. Attention was measured using the Forward Digit Span subtest (WAIS-IV) [24] and the Trail Making Test Part A (TMT-A) [25]. The Rey Auditory Verbal Learning Test [26] was used to assess Verbal Memory, specifically the first trial, immediate recall (sum of trials 1–5), and delayed recall subscales. Finally, executive function was tested with the Trail Making Test Part B (TMT-B) [25], the phonemic verbal fluency PMR test, adapted for Spanish speaking population [27, 28], the number of categories from the Wisconsin Card Sorting Test [29], and the Digit Symbol Substitution subtest (WAIS-IV) [24].

### Functional assessment

The psychosocial functioning was evaluated through the Functioning Assessment Short Test (FAST) [30, 31]. This scale was designed to detect functional difficulties in patients with mental disorders, specifically for bipolar disorder patients. FAST consists of 24 items that assess six specific areas of psychosocial functioning: (i) Autonomy refers to the patient’s ability to decide and carry out daily activities, (ii) Occupational functioning is related to the capacity to be employed as well as to maintain an adequate work performance, (iii) Cognitive functioning refers to the ability to concentrate, to learn and memorize information, and to solve problems, (iv) Financial issues are related to the ability to manage their own money properly, (v) Interpersonal relationships refers to the rapport with family, partners, and friends, involvement in social activities, having satisfactory sexual relationships, and the ability to defend one’s own interests, and (vi) Leisure time refers to doing sport or exercise and maintaining hobbies. All items are scored from 0 to 4, with higher scores suggesting greater functional disability. A previous study had determined that FAST total score from 12 to 20 indicated a mild impairment in some areas of psychosocial functioning and scores from 21 to higher represented moderate to severe impairment in most areas of functioning [31].

### Statistical analyses

Statistical analyses were carried out by means of the Statistical Package for Social Sciences, version 26. This study used the raw scores of the FAST total score as well as the different scores of the different functional subdomains that comprise this scale: autonomy, occupational, cognitive functioning, financial issues, interpersonal relationships, and leisure time. Three measures of the PDQ were also used, including in the study PDQ attention, PDQ memory (retrospective plus prospective), and PDQ executive function. To reduce the number of neuropsychological variables and following the recommendations to address cognitive impairment [32], three cognitive domains (Attention, Verbal Memory, and Executive Function) were defined by averaging *T*-scores of different neuropsychological tests mentioned above (by summing scores and dividing by the number of included tests).

A descriptive analysis of sociodemographic, clinical, functional, and cognitive measures was performed using absolute and relative frequency measures for categorical variables and calculating mean and standard deviation statistics for continuous variables. Differences between remitted and non-remitted patients were assessed by means of *t*-tests for continuous variables and chi-square tests for categorical variables. A series of multiple linear regression analyses were carried out to identify the independent factors associated with the FAST total and subdomains scores in remitted patients and in non-remitted patients, separately. To perform these linear regression analyses, Pearson correlations were used to examine bivariate relationships between FAST scores and the following variables: age, sex, years of schooling, estimated IQ, age at illness onset, depressive episodes, HDRS-17, Attention, Verbal Memory, Executive Function, PDQ Attention, PDQ Memory, and PDQ Executive. Variables associated with each FAST assessment at an *α* level of 0.05 in the bivariate analyses were considered candidate predictors of the corresponding FAST assessment and incorporated into the subsequent linear regression analysis, for each subsample separately. Multiple linear regression analyses were performed with a backward procedure. The estimated minimum sample size to detect a significant difference greater than or equal to 7 units [33], and a common standard deviation of 12, was 38 remitted subjects and 77 non-remitted subjects, accepting an alpha risk of 0.05 and a beta risk of 0.2 in a two-sided test.

## Results

### Participant characteristics and differences between remitted and non-remitted patients

Sociodemographic, clinical, functional, and cognitive characteristics of the patients at clinical remission (*n* = 117) and in non-remission (*n* = 208) are shown in Table 1. In detail, non-remitted patients showed fewer years of schooling as well as a lower estimated IQ. Likewise, non-remitted patients had a higher degree of depressive symptomatology than remitted patients as clinically expected due to grouping variable. At the level of psychosocial functioning, non-remitted patients indicated greater functional difficulties, not only in the overall functioning, but also in each of the different functional subdomains. In terms of objective cognitive performance, significant differences were observed between the two study groups, with higher scores shown in the remitted patients group. In subjective cognition, difference was observed in the self-perceived executive functioning, showing a greater perception of deficit in those non-remitted patients.

**Table 1.** Sociodemographic, clinical, functional, and cognitive characteristics between remitted and non-remitted patients

			Statistics	
	Remitted patients (*n* = 117)	Non-remitted patients (*n* = 208)	*t* or *χ* ^2^	*p*
Age, *years*	51.56 (9.73)	52.55 (7.69)	1.03	.312
Sex, female (*n*, %)	76 (65)	151 (72.6)	2.08	.150
Years of schooling, *n*	13.36 (3.88)	10.5 (3.65)	43.96	<.001
Estimated IQ, *T* score	55.16 (8.82)	49.75 (7.93)	31.95	<.001
Age at illness onset, *years*	37.39 (12.85)	40.03 (11.47)	3.62	.058
Depressive episodes, *n*	2.57 (1.52)	2.54 (1.53)	0.028	.868
HDRS-17, *total score*	4.04 (2.07)	17.96 (6.54)	500.37	<.001
FAST, *total score*	15.74 (9.67)	39.27 (14.25)	253.34	<.001
FAST autonomy	1.73 (1.99)	5.27 (3.33)	110.42	<.001
FAST occupational	4.16 (4.62)	11.1 (4.97)	153.08	<.001
FAST cognitive	4.36 (2.9)	9 (3.78)	132.97	<.001
FAST financial	0.43 (0.85)	1.53 (1.83)	37.89	<.001
FAST interpersonal	3.65 (2.91)	8.11 (4.03)	110.82	<.001
FAST leisure	1.79 (1.55)	3.85 (2.07)	88.11	<.001
Attention*, T* score	48.39 (7.48)	41.65 (7.79)	57.27	<.001
Verbal memory*, T* score	45.34 (8.63)	40.23 (9.63)	22.54	<.001
Executive function*, T* score	48.96 (6.15)	42.87 (7.37)	53.19	<.001
PDQ, *total score*	23.22 (13.63)	42.18 (13.94)	140.7	<.001
PDQ attention	10.07 (4.37)	10.8 (4.72)	1.89	.170
PDQ memory	14.22 (7.79)	15.59 (8.59)	2.02	.156
PDQ executive	5.95 (4.11)	11.82 (4.04)	156.14	<.001

Abbreviations: FAST, Functioning Assessment Short Test; HDRS-17, Hamilton Depression Rating Scale; IQ, intelligence quotient; *p*, significance level; PDQ, Perceived Deficit Questionnaire.

*Note*: Values represent mean scores (standard deviation) unless otherwise specified.

### Bivariate relationships of FAST total and subdomains scores with sociodemographic, clinical, and cognitive characteristics

Pearson’s correlation coefficients and *p*-values are presented in Table 2 for both, remitted and non-remitted patients. Those significant associations were to be included in the regression models.

**Table 2.** Pearson’s correlation coefficients (*p*-values) between FAST total and FAST subdomains and possible explanatory factors

		Age	Sex^a^	Years of schooling	Estimated IQ	Age at illness onset	Depressive episodes	HDRS-17	Attention	Verbal memory	Executive function	PDQ attention	PDQ memory	PDQ executive
**REMITTED PATIENTS**	FAST TOTAL	.166	.117	−.035	.084	−.132	**.277**	**.411**	−.107	−.162	**−.191**	−.029	−.118	**.565**
		(.074)	(.207)	(.711)	(.371)	(.155)	**(.003)**	**(<.001)**	(.254)	(.083)	**(.043)**	(.753)	(.206)	**(<.001)**
	FAST autonomy	.077	.007	−.081	.042	.012	.015	**.206**	−.024	.003	.002	.018	.028	**.411**
		(.407)	(.939)	(.385)	(.655)	(.898)	(.871)	**(.026)**	(.797)	(.977)	(.984)	(.847)	(.761)	**(<.001)**
	FAST occupational	**.205**	−.036	−.139	−.049	−.072	**.182**	**.360**	−.066	**−.196**	**−.212**	−.032	−.132	**.200**
		**(.026)**	(.697)	(.135)	(.605)	(.441)	**(.049)**	**(<.001)**	(.481)	**(.035)**	**(.024)**	(.734)	(.155)	**(.030)**
	FAST cognitive	.086	**.203**	−.061	.086	−.160	**.210**	**.277**	−.034	−.094	−.086	.117	.090	**.500**
		(.356)	**(.028)**	(.511)	(.359)	(.085)	**(.023)**	**(.002)**	(.719)	(.314)	(.366)	(.208)	(.335)	**(<.001)**
	FAST financial	−.114	.116	.073	−.039	−.114	.042	.111	.109	.084	.112	.126	.083	**.343**
		(.221)	(.212)	(.434)	(.679)	(.219)	(.650)	(.232)	(.245)	(.371)	(.236)	(.175)	(.375)	**(<.001)**
	FAST interpersonal	.092	.029	.149	.139	.038	.151	**.332**	−.053	−.123	−.032	−.037	−.096	**.329**
		(.325)	(.759)	(.110)	(.136)	(.684)	(.104)	**(<.001)**	(.573)	(.189)	(.738)	(.689)	(.303)	**(<.001)**
	FAST leisure	.078	−.020	−.009	.133	−.033	.144	**.238**	−.069	−.058	−.101	.020	−.049	**.459**
		(.406)	(.827)	(.926)	(.155)	(.725)	(.121)	**(.010)**	(.463)	(.538)	(.289)	(.831)	(.601)	**(<.001)**
**NON-REMITTED PATIENTS**	FAST TOTAL	.040	.016	**−.202**	**−.170**	.027	−.093	**.574**	**−.279**	**−.333**	**.−337**	−.055	−.016	**.544**
		(.564)	(.823)	**(.003)**	**(.014)**	(.698)	(.183)	**(<.001)**	**(<.001)**	**(<.001)**	**(<.001)**	(.427)	(.822)	**(<.001)**
	FAST autonomy	.106	−.063	**−.168**	−.068	.082	−.040	**.451**	**−.184**	**−.249**	**−.245**	−.085	−.019	**.473**
		(.129)	(.367)	**(.015)**	(.331)	(.236)	(.568)	**(<.001)**	**(.008)**	**(<.001)**	**(.001)**	(.221)	(.782)	**(<.001)**
	FAST occupational	.046	**−.142**	−.097	−.083	.004	.016	**.332**	**−.146**	**−.174**	**−.201**	.067	**.144**	**.270**
		(.507)	**(.040)**	(.165)	(.234)	(.958)	(.819)	**(<.001)**	**(.035)**	**(.012)**	**(.008)**	(.339)	**(.038)**	**(<.001)**
	FAST cognitive	.088	**.147**	**−.189**	**−.190**	.031	−.075	**.506**	**−.213**	**−.312**	**−.256**	−.047	−.055	**.526**
		(.208)	**(.034)**	**(.006)**	**(.006)**	(.659)	(.282)	**(<.001)**	**(.002)**	**(<.001)**	**(.001)**	(.499)	(.429)	**(<.001)**
	FAST financial	.080	−.017	**−.155**	**−.159**	.036	−.057	**.375**	**−.233**	**−.173**	**−.227**	−.136	−.066	**.389**
		(.252)	(.809)	**(.026)**	**(.022)**	(.602)	(.416)	**(<.001)**	**(.001)**	**(.013)**	**(.003)**	(.051)	(.343)	**(<.001)**
	FAST interpersonal	.093	.006	**−.151**	−.059	.020	−.103	**.524**	**−.217**	**−.198**	**−.257**	−.084	−.081	**.431**
		(.184)	(.929)	**(.030)**	(.397)	(.772)	(.138)	**(<.001)**	**(.002)**	**(.004)**	**(.001)**	(.229)	(.244)	**(<.001)**
	FAST leisure	−.034	.1	**−.144**	−.136	−.035	−.028	**.381**	**−.264**	**−.220**	**−.230**	.071	.117	**.437**
		(.627)	(.149)	**(.039)**	(.051)	(.615)	(.683)	**(<.001)**	**(<.001)**	**(.001)**	**(.002)**	(.311)	(.091)	**(<.001)**

Abbreviations: FAST, Functioning Assessment Short Test; HDRS-17, Hamilton Depression Rating Scale; IQ, intelligence quotient; PDQ, Perceived Deficit Questionnaire.

a Strickly speaking, the correlation coefficients appearing in this column correspond to point–biserial correlation values (the point–biserial correlation is, however, mathematically equivalent to the Pearson correlation).

Variables in bold were considered candidate predictors and were incorporated in the subsequent linear regression analysis

### Backward regression models predicting psychosocial functioning

The PDQ Executive was a significant independent factor in all the regression models run in the sample of remitted patients, except in the FAST Occupational model. HDRS-17 was found to be significant in the regression models of FAST total score, FAST Occupational, and FAST Interpersonal, while the Executive Function was only significant for the FAST Occupational model (data are shown in Table 3).

**Table 3.** Backward regression models to assess the relationship between possible explanatory factors and functioning measures remitted patients

Outcome measures	Regressors	Statistics	*F*	*df*	*p*	*R* ^2^
FAST total	**Depressive episodes**	*β* = .80, *p* = .092, RW = .041	19.25	4, 108	<.001	.427
	**HDRS-17**	*β* = 1.01, *p* = .004, RW = .105				
	**Executive function**	*β* = −.21, *p* = .073, RW = .026				
	**PDQ executive**	*β* = 1.09, *p* < .001, RW = .254				
FAST autonomy	HDRS-17		23.41	1, 115	<.001	.169
	**PDQ executive**	*β* = .2, *p* < .001, RW = .169				
FAST occupational	**Age**	*β* = .08, *p* = .051, RW = .034	8.27	3, 109	<.001	.195
	Depressive episodes					
	**HDRS-17**	*β* = .64, *p* = .002, RW = .113				
	Verbal memory					
	**Executive function**	*β* = −.17, *p* = .010, RW = .048				
	PDQ executive					
FAST cognitive	Sex		21.07	2, 114	<.001	.269
	Depressive episodes					
	**HDRS-17**	*β* = .21, *p* = .079, RW = .048				
	**PDQ executive**	*β* = .32, *p* < .001, RW = .221				
FAST financial	**PDQ executive**	*β* = .07, *p* < .001, RW = .117	15.29	1, 115	<.001	.117
FAST interpersonal	**HDRS-17**	*β* = .37, *p* = .004, RW = .086	11.73	2, 114	<.001	.171
	**PDQ executive**	*β* = .18, *p* = .005, RW = .084				
FAST leisure	HDRS-17		30.64	1, 115	<.001	.210
	**PDQ executive**	*β* = .17, *p* < .001, RW = .210				

Abbreviations: *β*, beta; *df*, degrees of freedom; *F*, *F* test; FAST, Functional Assessment Short Test; HDRS-17, Hamilton Depression Rating Scale; *p*, significance level; PDQ, Perceived Deficit Questionnaire; *R* ^2^, variance explained; RW, raw relative weight.

*Note*: The regressors of the final model are marked in bold with their corresponding parameter estimates.

**Figure 1.**
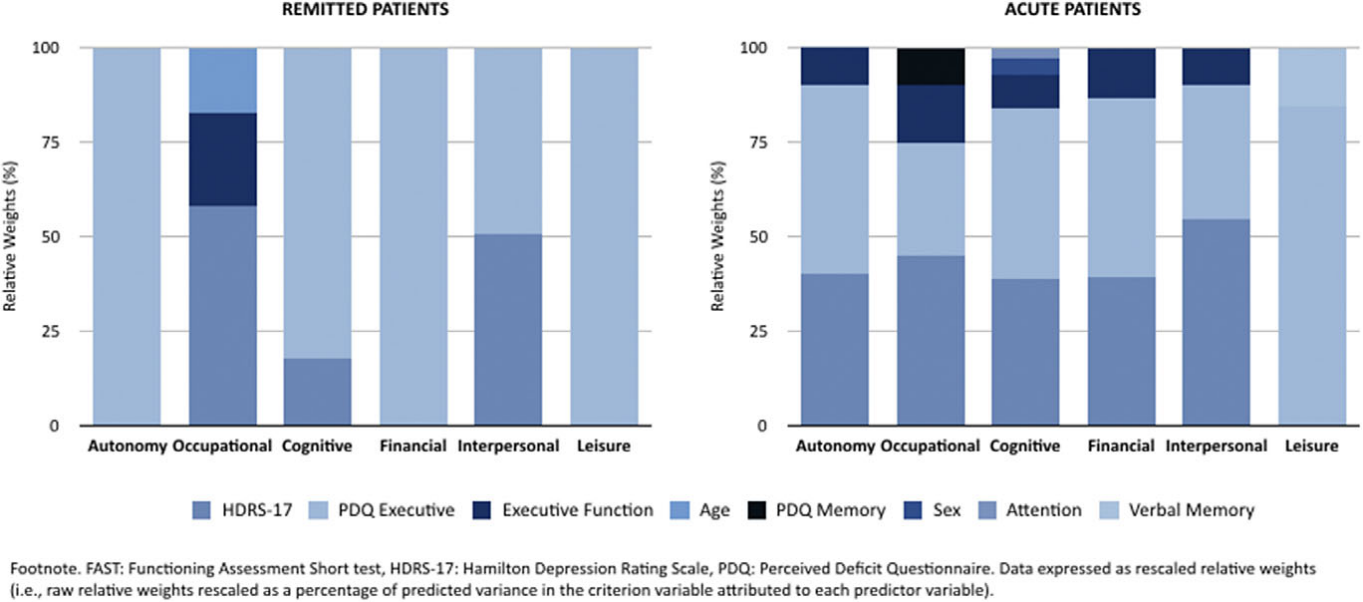
Relative importance of sociodemographic, clinical, and cognitive characteristics for predicting psychosocial functioning (FAST scores).

In non-remitted patients, the significant factors were PDQ Executive and HDRS-17 in all of the regression models (except HDRS-17 in the FAST Leisure model). Executive function was observed in most regression models; however, it reached statistical significance only in specific cases, such as the FAST total score, FAST Cognitive, and FAST Financial models. The FAST Occupational model also included PDQ Memory as a significant factor, as well as verbal memory in the FAST Leisure model and being female in the FAST Cognitive model (data are shown in Table 4). A graphical representation of variable contributions to regression effects can be found in Figure 1.

**Table 4.** Backward regression models to assess the relationship between possible explanatory factors and functioning measures in non-remitted patients

Outcome measures	Regressors	Statistics	*F*	*df*	*p*	*R* ^2^
FAST total	Years of schooling		53.13	3, 169	<.001	.521
	Estimated IQ					
	**HDRS-17**	*β* = .88, *p* < .001, RW = .229				
	Attention					
	Verbal memory					
	**Executive function**	*β* = −.36, *p* = .001, RW = .071				
	**PDQ executive**	*β* = 1.34, *p* < .001, RW = .221				
FAST autonomy	Years of schooling		28.48	3, 169	<.001	.336
	**HDRS-17**	*β* = .16, *p* < .001, RW = .139				
	Attention					
	Verbal memory					
	**Executive function**	*β* = −.06, *p* = .053, RW = .035				
	**PDQ executive**	*β* = .30, *p* < .001, RW = .173				
FAST occupational	Sex		8.95	4, 168	<.001	.171
	**HDRS-17**	*β* = .2, *p* = .001, RW = .077				
	Attention					
	Verbal memory					
	**Executive function**	*β* = −.81, *p* = .094, RW = .026				
	**PDQ memory**	*β* = .09, *p* = .034, RW = .051				
	**PDQ executive**	*β* = .19, *p* = .037, RW = .017				
FAST cognitive	**Sex**	*β* = 1.47, *p* = .003, RW = .021	25.03	5, 167	<.001	.463
	Years of schooling					
	Estimated IQ					
	**HDRS-17**	*β* = .21, *p* < .001, RW = .179				
	**Attention**	*β* = .08, *p* = .069, RW = .013				
	Verbal memory					
	**Executive function**	*β* = −.13, *p* = .006, RW = .04				
	**PDQ executive**	*β* = .38, *p* < .001, RW = .210				
FAST financial	Years of schooling		14.18	3, 169	<.001	.201
	Estimated IQ					
	**HDRS-17**	*β* = .05, *p* = .006, RW = .095				
	Attention					
	Verbal memory					
	**Executive function**	*β* = −.03, *p* = .04, RW = .032				
	**PDQ executive**	*β* = .13, *p* < .001, RW = .116				
FAST interpersonal	Years of schooling		28.73	3, 169	<.001	.338
	**HDRS-17**	*β* = .26, *p* < .001, RW = .204				
	Attention					
	Verbal memory					
	**Executive function**	*β* = −.07, *p* = .053, RW = .038				
	**PDQ executive**	*β* = .28, *p* < .001, RW = .132				
FAST leisure	Years of schooling		28.77	2, 170	<.001	.209
	HDRS-17					
	Attention					
	**Verbal memory**	*β* = −.04, *p* = .004, RW = .033				
	Executive function					
	**PDQ executive**	*β* = .21, *p* < .001, RW = .176				

Abbreviations: *β*, beta; *df*, degrees of freedom; *F*, *F* test; FAST, Functional Assessment Short Test; HDRS-17, Hamilton Depression Rating Scale; *p*, significance level; PDQ, Perceived Deficit Questionnaire; *R* ^2^, variance explained; RW, raw relative weight.

*Note*: The regressors of the final model are marked in bold with their corresponding parameter estimates.

## Discussion

This study identifies the factors that are independently associated with the distinct facets of psychosocial functioning at different stages of major depression. Depressive symptoms, executive difficulties, and subjective executive complaints appear to be the significant factors of functional outcomes in non-remitted patients, while only residual depressive symptoms and self-appraisal of executive competence are significantly associated with psychosocial functioning in remitted patients. Interestingly, these factors are common predictors of the different subdomains of FAST in both non-remitted and remitted patients with MDD. Considering that in the last decade, the treatment goal of depression has evolved from exclusively trying to reduce depressive symptomatology to improve patients’ functioning [4, 15], identifying which clinical and cognitive variables are related to this functional recovery may be an essential aim in the intervention design.

One clear fact that emerges from the present data is the clinical value of the patient’s appraisal of their own cognitive functioning. The use of subjective measures of cognition in clinical practice has been proposed as part of the assessment of patients’ cognitive complaints. Yet studies attempting to correlate these subjective measures with neuropsychological tests results have not been entirely successful as there is a significant discrepancy between those measures [34–36]. These discordances have given rise to doubts about their use in clinical routine. One possible explanation for this lack of agreement may be the fact that subjective and objective cognitive measures do not assess the same capabilities. In other words, the neuropsychological test is done in a controlled context and without distracting stimuli, whereas the subjective assessments reflect everyday situations in which there are a multitude of stimuli, as well as interaction with other people, that can influence their performance. Therefore, it is reasonable to think about a possible direct relationship between subjective measures of cognition with the daily functioning as observed in the present study. However, there are not as many studies that relate subjective measures of cognition to psychosocial functioning in depression, and even fewer which are specific as to which subjective cognitive domain determines this relationship. Regardless of the few that exist, overall perceived cognitive impairment is known to be associated with worse functional outcomes [34, 37–39] as it is also seen in the current findings. These results highlight the relevance of counting with what patients believe or think about their cognitive competence in clinical practice, as these beliefs or thoughts may determine what they do to regain an effective level of functioning. According to the current results, the perception of cognitive deficits is limited to self-appraised difficulties in executive function, with no apparent association with attention or memory. This finding suggests that PDQ questions directed at one’s perception of organization, planning, and problem-solving skills are more directly related to psychosocial functioning itself, whatever the depressive symptomatology is. In contrast, items related to attention or memory do not appear to exert a direct impact on patients’ functioning. This could be explained by the fact that these items describe cognitive aspects more closely aligned with the constructs assessed by neuropsychological tests. Another possibility is that the PDQ scale might not have been developed entirely from the patient’s perspective and it may not be identifying the most common attentional and memory difficulties in patients with depression [40, 41]. Nevertheless, more research is needed to understand what role self-perceived cognitive performance plays in everyday life.

It is important to bear in mind that not only subjective cognitive appraisal but also the objective cognitive impairment has a relevant role in determining daily functioning of patients with MDD. There is some empirical evidence that cognitive impairment (such as deficits in attention and processing speed, executive function, and verbal learning) mediates functional outcomes in depression [6, 42–44]. In the current study, in non-remitted patients, objective cognition measures remain clearly associated with worse psychosocial functioning, along with subjective cognition and clinical severity. Specifically, objective executive function is the only cognitive domain that is associated with an overall psychosocial functioning, and with the different areas of functioning (with the exception of FAST leisure), in agreement with previous research [6, 42, 43].

According to foregoing scientific literature, executive functioning also plays a role in the degree of functional impairment of patients in clinical remission, and it determines functioning in specific functional areas such as in occupational functioning, in subjective cognition, and in leisure time [6]. However, the results of the present study contradict such previous findings since the perceived cognitive functioning seems to be the only cognitive determinant in full clinical remission. A possible explanation could be that remitted patients showed better executive functioning when explored with objective tests, and thus no relation could be found with different psychosocial subdomains. In this same line, two previous studies have observed a stronger correlation between subjective cognition to psychosocial functioning rather than with objective cognitive variables [34, 37], and they have also confirmed the importance of subjective cognitive performance in the restoration of daily activities once the depressive episode is resolved. It is relevant to note that different characteristics such as demographics related to sex and socioeconomic status together with the symptom severity across study samples, or the cognitive domains used in previous research, as well as the grouping based on patients’ cognitive profiles (ranging from global impairment to mild or no impairment), might have interfered with the understanding of the relationship between cognition (either objective or subjective) and psychosocial functioning [16, 45–48]. Being female seemed to play a discrete role in FAST cognitive results, indicating that gender perspective should be taken into consideration in future studies that address cognitive, psychosocial, and clinical outcomes altogether.

It must be pointed out that the present study has not only assessed which variables are the most related to functional outcomes, but also which factors are related to each of the different areas that constitute a person’s overall functioning. In fact, it is observed that depressive symptomatology, executive function, and the patient’s perception of memory and executive functioning influence occupational functioning in non-remitted patients, whereas when the patient is in clinical remission, only residual depressive symptomatology and the objective measure of executive functioning are determinants. These results underline the need of executive skills to function properly at work at all stages of the disease. In other words, achieving good executive performance seems to be of utmost importance to reach optimal occupational functioning.

The present findings suggest that in order to achieve a full functional recovery, psychotherapeutic interventions should not only aim at improving psychosocial functioning through cognitive enhancement, but it should also take into account the patient’s perception of their cognitive functioning, especially in those stages of greater clinical stability in which the greatest predictor of psychosocial functioning will be the patient’s own perception of their executive abilities. In clinical practice, it is observed that there is a profile of patients who, despite being in clinical remission, have a significant number of complaints about not having recovered their previous level of functioning, as if certain executive skills have not been recovered and make it difficult for patients to carry out day-to-day activities. Consequently, it may be necessary to develop strategies to better manage their self-perception, and not only interventions directed at cognitive training.

Nonetheless, there are some limitations worth mentioning that might affect the understanding of the observed results. First, some clinical variables as anxiety, level of self-efficacy, personality traits, or the depressive symptomatology itself might influence the patient’s perceived cognition [21], and in turn, these variables might impact on patients’ psychosocial functioning. It is possible that the fact that the self-appraisal in organization and planning skills that remain significant in clinical remission may be due to the presence of those psychological characteristics. Second, the subjective cognition measurement scale itself (PDQ) was not initially developed for depression and, although it was subsequently adapted and validated for this population [23], it may not fully represent the most common cognitive difficulties in depressed patients, especially in the domains of attention and memory. Third, the psychosocial functioning scale (FAST) was created for bipolar disorder and has not been validated for the depressive population, so it may not reflect the functional difficulties encountered by patients with depression. Fourth, the FAST scale is only validated as a total outcome measure, and not designed to consider the different functional areas that comprise it. However, previous studies [6] have used the different functional subdomains as part of the statistical analysis because of the great clinical interest in knowing exactly what is affected in depressed patients, in order to target interventions to these particular difficulties. Fifth, given the lack of an observable measure of psychosocial functioning, the current findings may have been influenced by the subjective appraisal itself. Finally, only two stages of the disease (remission vs. non-remission) are being considered, and unfortunately not all clinical scenarios are being represented in terms of the psychosocial functioning of patients. Due to the design of the study is not possible to analyse every depression stage as well as the potential variables that could be influencing the outcomes.

In conclusion, this study provides evidence on the relevance of patients’ self-awareness of their cognitive functioning in carrying out daily activities, from work performance to interpersonal relationships. These findings have significant implications for the clinical management of patients MDD. It highlights the importance of incorporating cognitive tests (both subjective and objective) and psychosocial functioning assessments into routine clinical practice. These aspects are often overlooked but are crucial for a comprehensive understanding of each individual’s condition. By integrating these assessments, clinicians can identify key factors that may hinder treatment adherence, impede finding the most suitable treatment for each patient, and ultimately prevent some individuals from achieving full recovery. Consequently, it is important to continue researching on this issue to develop interventions aimed at achieving full recovery [49], which are based not only on reducing depressive symptomatology, but also on improving objective cognition, specially including patients’ appraisal of their own cognitive functioning.

## Data Availability

The data that support the findings of this study are available from the corresponding author upon reasonable request.
